# Resilience, Coping Strategies, and Trauma-Related Symptoms in the Mexican Population Diagnosed With Obsessive-Compulsive Disorder

**DOI:** 10.7759/cureus.88939

**Published:** 2025-07-28

**Authors:** Carla Brueggerhoff-Batel, Cristina Lóyzaga-Mendoza, Héctor Guzmán-González

**Affiliations:** 1 Department of Education, National Institute of Psychiatry, Mexico City, MEX; 2 Obsessive Compulsive Disorder and Obsessive Spectrum Clinic, National Institute of Psychiatry, Mexico City, MEX

**Keywords:** coping strategies, obsessive-compulsive disorder, ptsd symptoms, resilience, trauma

## Abstract

Introduction: Obsessive-compulsive disorder (OCD) is characterized by the presence of obsessions and compulsions, which are performed in an attempt to alleviate anxiety. It is imperative to identify additional factors that may influence symptom severity and treatment efficacy. This study aims to evaluate the relationship among resilience, coping strategies, and trauma with the severity of symptoms in the Mexican population diagnosed with OCD.

Methods: A prospective cross-sectional study was conducted at the National Institute of Psychiatry in Mexico City. The sample comprised 45 patients diagnosed with OCD. All participants underwent a clinical interview and completed the following instruments: the Yale-Brown Obsessive Compulsive Scale (Y-BOCS), the Brief Resilience Scale (BRS), the Coping Strategy Indicator (CSI), and the Post-Traumatic Stress Disorder Checklist for DSM-5 (PCL-5). Spearman’s rank correlation was performed to assess the relationship between the Y-BOCS scores and the scores obtained from the other scales. Furthermore, a subgroup analysis was performed based on the participants' gender.

Results: A weak negative correlation was observed between the BRS score and the severity of the Y-BOCS obsessive factor (r_s_ (43) = -0.31, p = 0.041), as well as between the CSI seeking social support score and the Y-BOCS compulsive factor (r_s_ (43) = -0.29, p = 0.050). Additionally, a weak positive correlation was identified between the CSI avoidance score and the Y-BOCS obsessive factor (r_s_ (43) = 0.30, p = 0.043), as well as between the total PCL-5 score and the Y-BOCS obsessive factor (r_s_ (43) = 0.30, p = 0.044). No significant associations were found between the scores of the BRS, CSI, and PCL-5 scales and the severity of the Y-BOCS when considering gender differences.

Conclusion: This study identified four key factors associated with the severity of OCD: resilience level, seeking social support as a coping strategy, avoidance behaviors, and trauma-related symptoms. These findings highlight additional elements that may influence patients’ treatment responses, quality of life, and overall prognosis.

## Introduction

Obsessive-compulsive disorder (OCD) is characterized by the presence of obsessions (intrusive and recurrent thoughts, urges, or images) and compulsions (repetitive and ritualized behaviors or mental acts), which are performed in an attempt to alleviate anxiety [1]. These symptoms cause significant distress and negatively impact the individual’s overall functioning [2]. The worldwide lifetime prevalence of OCD ranges from 1.9% to 2.5% [3]. In Mexico, the sole published study concerning the epidemiology of OCD, conducted in 2004, reported a lifetime prevalence of 1.4%, with a higher prevalence among women (1.7%) compared to men (0.8%) [4]. OCD often tends toward chronicity, with remission rates estimated at approximately 20% of patients, while 49% continue to experience significantly persistent symptoms over a 20-year follow-up period [5]. This chronicity results in substantial costs associated with OCD treatment; for instance, a study in the United Kingdom (UK) found an average annual treatment cost of 525 pounds (approximately 13,204 Mexican pesos) per person with OCD [6]. Although no study has examined the economic impact of OCD in Mexico, the costs reported in the UK suggest a significant burden for this population.

Research indicates that several factors significantly influence the persistence and severity of OCD symptoms, including resilience, coping strategies, and the history of a traumatic event in life [7-9]. Resilience is defined as a dynamic process through which individuals adapt positively to adverse experiences [10]. In patients diagnosed with OCD, resilience has been proposed as a determinant in modulating the stress response associated with obsessions and compulsions. One study found that lower levels of resilience were associated with greater OCD severity and anxiety compared to those individuals exhibiting higher levels of resilience [7]. Moreover, additional research suggests that treatment with selective serotonin reuptake inhibitors (SSRIs) may enhance resilience and cognitive flexibility in patients with OCD [11].

Coping represents a critical variable in the study of resilience, encompassing both cognitive and behavioral efforts aimed at managing, tolerating, or alleviating external and internal demands that are perceived as stressful [12]. This construct can be categorized into two primary types: active coping strategies and avoidance-based coping strategies. Active strategies include cognitive reappraisal, seeking social support, and taking concrete actions. In contrast, avoidance mechanisms involve distraction, ignoring the situation, minimizing the issue, withdrawing, escaping, or engaging in wishful thinking [13]. Notably, individuals diagnosed with OCD exhibit a greater tendency to employ avoidance-based coping mechanisms, which are also associated with the severity of the disorder [8].

Furthermore, a meta-analysis found that a history of a traumatic event in patients diagnosed with some obsessive-compulsive spectrum disorder, including OCD, is correlated with heightened symptom severity, particularly regarding compulsions and in women. Interpersonal trauma (emotional abuse, violence, sexual abuse, and neglect) has been identified as the predominant type of trauma associated with the severity of OCD. The association between trauma and OCD may be attributed to the emergence of intrusive and recurrent thoughts that evoke the traumatic experience, as well as the subsequent development of repetitive behaviors intended to mitigate anxiety as a coping mechanism [9]. This observation aligns with the comorbid presence of post-traumatic stress disorder (PTSD) in 19.1% of patients diagnosed with OCD [14]. Additionally, research has indicated that a predominance of avoidance-based coping strategies is associated with a higher likelihood of developing PTSD [15].

Considering the chronic nature of this disorder, the significant costs associated with its treatment, and the limited information available in the Mexican population, it is essential to identify additional factors that may influence the severity of symptoms and the effectiveness of both pharmacological and psychotherapeutic interventions. This study aims to evaluate the relationship among resilience, coping strategies, and trauma with the severity of symptoms in a sample of the Mexican population diagnosed with OCD. We hypothesize that patients will demonstrate low levels of resilience and a greater inclination to utilize avoidance-based coping strategies, which will be correlated with the severity of OCD. Similarly, an association will be found between trauma-related symptoms and the severity of OCD, particularly among women.

## Materials and methods

Participants

This prospective cross-sectional study was conducted from December 2024 to April 2025, involving patients receiving care at the Obsessive-Compulsive Disorder and Obsessive Spectrum Clinic of the National Institute of Psychiatry, the primary referral center for specialized OCD treatment in Mexico City. For the recruitment of participants, a non-probabilistic convenience sampling method was employed. The study included men and women aged 18 to 70 years diagnosed with OCD and who had received pharmacological treatment for more than three months. Patients diagnosed with a psychotic spectrum disorder or those identified as being at risk for suicide during the time of evaluation were excluded from participation.

Study procedures

A clinical interview, lasting approximately 60 minutes, was conducted by a trained psychiatrist, during which sociodemographic and clinical data were collected. Additionally, various instruments were employed to evaluate the severity of OCD, resilience, coping strategies, and trauma-related symptoms.

OCD assessment

Obsessive-compulsive symptoms (OCS) were evaluated using the Yale-Brown Obsessive Compulsive Scale (Y-BOCS), which is recognized as the gold standard for assessing both the presence and severity of OCS [16]. This semi-structured scale, administered by a clinician, features a severity scale that appraises time spent, interference caused, distress experienced, resistance, and control exerted over obsessions and compulsions. The scale comprises 10 items, each of which is scored from 0 to 4 [17]. Scores ranging from 0 to 7 represent subclinical symptoms, 8 to 15 illustrate mild symptoms, 16 to 23 express moderate symptoms, 24 to 31 denote severe symptoms, and 32 to 40 imply extreme symptoms [16]. In the Mexican population, this scale has exhibited good validity, with Cronbach’s alpha of 0.85 [18].

Resilience assessment

The degree of resilience was measured using the Brief Resilience Scale (BRS), a six-item self-report instrument designed to assess resilience through a series of statements related to personal characteristics. Respondents rate each item on a scale from 1 (completely disagree) to 5 (completely agree). The total score is calculated by summing the responses and dividing by 6. The ratings are categorized as follows: a score of 1 to 2.99 indicates low resilience, 3 to 4.30 signifies normal resilience, and 4.31 to 5 reflects high resilience. The original scale demonstrates good internal consistency, with Cronbach’s alpha values ranging from 0.81 to 0.91 [19]. The Spanish version exhibits adequate internal consistency, with Cronbach’s alpha of 0.83 [20].

Coping strategy assessment

The Coping Strategy Indicator (CSI) was utilized to evaluate the coping strategies employed by participants in stressful situations. This self-report scale comprises three subscales, each consisting of 11 items that assess problem-solving, seeking social support, and avoidance. Participants rate each item on a scale from 1 (not at all) to 3 (a lot). The total score from each subscale reflects the degree to which each coping strategy is utilized. The CSI demonstrates adequate internal consistency, with Cronbach’s alpha values of 0.92 for seeking social support, 0.89 for problem-solving, and 0.83 for avoidance [21].

PTSD symptom assessment

PTSD symptoms were measured using the Post-Traumatic Stress Disorder Checklist for DSM-5 (PCL-5). This self-report instrument comprises 20 items that evaluate PTSD. Each item is rated on a Likert scale ranging from 0 (not at all) to 4 (extremely), resulting in a total score ranging from 0 to 80. A cut-off score between 31 and 33 indicates probable PTSD [22]. The scale demonstrates excellent internal consistency, with Cronbach’s alpha of 0.94 [23]. Furthermore, it has been validated for the Mexican population with Cronbach’s alpha of 0.97 [24].

Statistical analysis

In this analysis, categorical variables were represented by frequencies and percentages, while continuous variables were expressed as medians with interquartile ranges (IQRs). The Shapiro-Wilk test was employed to evaluate the normality of the data. Since the Shapiro-Wilk test indicated a lack of normality, Spearman’s rank correlation was performed to assess the relationship between variables. The sample size provided a statistical power of 0.94 for these correlations. For subgroup analyses based on participant gender, categorical variables were evaluated using either the χ² test or Fisher’s exact test, while the Mann-Whitney U test was employed for continuous variables. A significance level of p ≤ 0.05 was established for all tests. All statistical analyses were performed using Jamovi 2.6.44.0 for macOS.

Ethical considerations

The study was approved by the Ethics Committee of the National Institute of Psychiatry, with the approval number CEI/C/053/2024. All the individuals provided written informed consent to participate in the research. To ensure confidentiality, personal data were recorded using an alphanumeric code. The authors have no conflicts of interest to declare.

## Results

Sociodemographic characteristics

The study comprised a total of 45 participants, including 23 (51.11%) men and 22 (48.89%) women. The median age of the participants was 31 (IQR = 19.00) years. Among them, 11 (24.44%) were in a relationship, while 34 (75.56%) identified as single. In terms of employment status, 36 (80.00%) participants were employed, whereas nine (20.00%) were unemployed. The median educational attainment was 12.00 (IQR = 0.00) years.

OCD (Y-BOCS score)

The median severity of OCD, as assessed by the Y-BOCS, was 9.00 (IQR = 6.00) for obsessions and 9.00 (IQR = 8.00) for compulsions, resulting in a median total score of 18.00 (IQR = 14.00), representing moderate OCD severity. The most prevalent OCD subtype was aggressive, affecting 17 (37.78%) individuals, followed by the contamination subtype in 16 (35.56%) participants. Other subtypes included order and symmetry in three (6.67%), religious in three (6.67%), somatic in three (6.67%), superstitious in two (4.44%), and sexual in one (2.22%) participant. Additionally, 17 (37.78%) of the included participants had previously undergone Cognitive Behavioral Therapy with Exposure and Response Prevention (CBT-ERP). Further clinical characteristics are shown in Table 1.

**Table 1:** Sociodemographic and clinical characteristics. BRS: Brief Resilience Scale; CBT-ERP: Cognitive Behavioral Therapy with Exposure and Response Prevention; CSI: Coping Strategy Indicator; IQR: interquartile range; OCD: obsessive-compulsive disorder; PCL-5: Post-Traumatic Stress Disorder Checklist for DSM-5; Y-BOCS: Yale-Brown Obsessive Compulsive Scale; U: Mann-Whitney U test *Time in years

	Total (n = 45)		Men (n = 23)		Women (n = 22)			
	n	%	n	%	n	%	χ^²^	p
Relationship	11	24.44	5	21.74	6	27.27	0.186	0.666
CBT-ERP	17	37.78	10	43.48	7	31.82	0.650	0.420
	n	%	n	%	n	%	Fisher’s exact test	p
Employment	36	80.00	20	86.96	16	72.73	-	0.284
	Median	IQR	Median	IQR	Median	IQR	U	p
Age*	31.00	19.00	27.00	19.00	39.50	17.00	162.000	0.040
Education*	12.00	0.00	12.00	4.00	12.00	0.00	222.000	0.437
Age at onset*	15.00	9.00	15.00	5.50	18.50	15.50	163.000	0.042
Age at first psychiatric assessment*	25.00	21.00	21.00	10.50	31.00	21.25	152.500	0.023
Time of untreated OCD*	10.00	12.00	9.00	9.00	10.00	12.50	223.000	0.502
Obsession (Y-BOCS)	9.00	6.00	7.00	6.50	9.50	4.75	177.000	0.086
Compulsion (Y-BOCS)	9.00	8.00	8.00	6.50	9.50	5.00	174.000	0.073
Total (Y-BOCS)	18.00	14.00	15.00	14.50	20.00	10.75	172.000	0.067
Problem-solving (CSI)	24.00	12.00	20.00	10.50	27.00	9.75	162.500	0.040
Seeking social support (CSI)	18.00	17.00	17.00	17.00	19.50	17.00	239.500	0.767
Avoidance (CSI)	25.00	5.00	24.00	6.00	25.00	4.75	211.500	0.350
BRS	3.10	0.70	3.10	0.50	3.20	0.85	246.000	0.882
PCL-5	12	17.00	9.00	12.50	16.00	21.50	208.000	0.312

Resilience (BRS score)

The median score of the BRS was 3.10 (IQR = 0.70). Based on these scores, participants were categorized into three distinct resilience groups: 12 (26.67%) individuals were identified as having low resilience, 31 (68.89%) exhibited normal resilience, and two (4.44%) demonstrated high resilience (Table 2).

**Table 2:** Interpretation of BRS, PCL-5, and Y-BOCS scores. BRS: Brief Resilience Scale; PCL-5: Post-Traumatic Stress Disorder Checklist for DSM-5; PTSD: post-traumatic stress disorder; Y-BOCS: Yale-Brown Obsessive Compulsive Scale *Fisher’s exact test was conducted to assess the differences between variables

	Total (n = 45)		Men (n = 23)		Women (n = 22)		
	n	%	n	%	n	%	p*
Y-BOCS							
Subclinical	9	20.00	6	26.09	3	13.64	0.240
Mild	10	22.22	7	30.43	3	13.64	
Moderate	17	37.78	8	34.78	9	40.91	
Severe	7	15.56	2	8.70	5	22.73	
Extreme	2	4.44	0	0.00	2	9.09	
BRS							
Low	12	26.67	5	21.74	7	31.82	0.457
Normal	31	68.89	16	69.57	15	68.18	
High	2	4.44	2	8.70	0	0.00	
PCL-5							
Probable PTSD	6	13.33	2	8.70	4	18.18	0.414

Coping strategies (CSI score)

The median scores of the CSI factors were as follows: 24.00 (IQR = 12.00) for problem-solving, 18.00 (IQR = 17.00) for seeking social support, and 25.00 (IQR = 5.00) for avoidance.

PTSD symptoms (PCL-5 score)

The median score of the PCL-5 was 12.00 (IQR = 17.00). Utilizing a cut-off score of 31 points, six (13.33%) participants were identified as having probable PTSD (Table 2).

Correlations between BRS, CSI, PCL-5, and Y-BOCS scores

Brief Resilience Scale

A weak negative correlation was observed between the BRS score and the severity of the Y-BOCS obsessive factor (r_s_ (43) = -0.31, p = 0.041).

Coping Strategy Indicator

A weak negative correlation was identified between the seeking social support score and the Y-BOCS compulsive factor (r_s_ (43) = -0.29, p = 0.050). Furthermore, a weak positive correlation was established between the avoidance score and the Y-BOCS obsessive factor (r_s_ (43) = 0.30, p = 0.043).

Post-Traumatic Stress Disorder Checklist for DSM-5

A weak positive correlation was identified between the total PCL-5 score and the Y-BOCS obsessive factor (r_s_ (43) = 0.30, p = 0.044) (Table 3).

**Table 3:** Correlation matrix of BRS, CSI, PCL-5, and Y-BOCS scores. Spearman’s rank correlation was performed to assess the relationship between variables. BRS: Brief Resilience Scale; CSI: Coping Strategy Indicator; PCL-5: Post-Traumatic Stress Disorder Checklist for DSM-5; Y-BOCS: Yale-Brown Obsessive Compulsive Scale *p < 0.05; **p < 0.01; ***p < 0.001

Variable	1	2	3	4	5	6	7
1. Obsession (Y-BOCS)							
2. Compulsion (Y-BOCS)	0.89***						
3. Total (Y-BOCS)	0.96***	0.98***					
4. Problem-solving (CSI)	0.00	-0.01	0.01				
5. Seeking social support (CSI)	-0.28	-0.29*	-0.28	0.48***			
6. Avoidance (CSI)	0.30*	0.16	0.23	0.07	-0.18		
7. BRS	-0.31*	-0.25	-0.25	0.50***	0.43**	-0.09	
8. PCL-5	0.30*	0.16	0.23	-0.21	-0.12	0.17	-0.05

Subgroup analysis (gender differences)

Upon examining the gender of the participants, it was observed that women exhibited a greater age (U = 162.000, p = 0.040), a later age at the onset of the disorder (U = 163.000, p = 0.042), an older age at first psychiatric evaluation (U = 152.500, p = 0.023), and higher scores on the problem-solving factor of the CSI (U = 162.500, p = 0.040) compared to men (Table 1). No associations were found between the BRS, CSI, and PCL-5 scores in relation to the Y-BOCS factors (Tables 4, 5). Additionally, no significant differences were noted in the levels of resilience (p = 0.457) and the presence of probable PTSD (p = 0.414) between men and women as assessed by Fisher’s exact test (Table 2).

**Table 4:** Correlation matrix of BRS, CSI, PCL-5, and Y-BOCS scores in women. Spearman’s rank correlation with Holm correction was performed to assess the relationship between variables. BRS: Brief Resilience Scale; CSI: Coping Strategy Indicator; PCL-5: Post-Traumatic Stress Disorder Checklist for DSM-5; Y-BOCS: Yale-Brown Obsessive Compulsive Scale *p < 0.05; **p < 0.01; ***p < 0.001

Variable	1	2	3	4	5	6	7
1. Obsession (Y-BOCS)							
2. Compulsion (Y-BOCS)	0.90***						
3. Total (Y-BOCS)	0.96***	0.97***					
4. Problem-solving (CSI)	-0.14	-0.23	-0.18				
5. Seeking social support (CSI)	-0.35	-0.37	-0.36	0.40			
6. Avoidance (CSI)	0.32	0.24	0.26	-0.10	-0.33		
7. BRS	-0.48	-0.47	-0.41	0.34	0.45	-0.31	
8. PCL-5	0.22	0.00	0.11	-0.45	-0.13	0.05	-0.13

**Table 5:** Correlation matrix of BRS, CSI, PCL-5, and Y-BOCS scores in men. Spearman’s rank correlation with Holm correction was performed to assess the relationship between variables. BRS: Brief Resilience Scale; CSI: Coping Strategy Indicator; PCL-5: Post-Traumatic Stress Disorder Checklist for DSM-5; Y-BOCS: Yale-Brown Obsessive Compulsive Scale *p < 0.05; **p < 0.01; ***p < 0.001

Variable	1	2	3	4	5	6	7
1. Obsession (Y-BOCS)							
2. Compulsion (Y-BOCS)	0.88***						
3. Total (Y-BOCS)	0.96***	0.96***					
4. Problem-solving (CSI)	-0.10	-0.04	-0.04				
5. Seeking social support (CSI)	-0.28	-0.26	-0.25	0.55			
6. Avoidance (CSI)	0.21	-0.01	0.13	0.19	-0.10		
7. BRS	-0.17	-0.05	-0.08	0.69***	0.44	0.16	
8. PCL-5	0.35	0.26	0.32	-0.04	-0.30	0.26	0.07

## Discussion

This study aimed to investigate how resilience, coping strategies, and trauma exposure relate to OCD symptom severity in the Mexican population. Our analysis found that several factors, including resilience levels, coping strategies like seeking social support and avoidance behaviors, and trauma-related symptoms, are associated with the severity of OCD symptoms.

Resilience

First, we identified a weak negative correlation between resilience levels and the severity of the Y-BOCS obsessive factor. This finding is consistent with a previous study conducted during the COVID-19 pandemic, which indicated that lower resilience predicted more severe OCD symptoms at a six-month follow-up [7]. Furthermore, a recent study conducted in Egypt confirmed a negative correlation between resilience and the total Y-BOCS score [25]. Notably, our results suggest that the severity of obsessions is particularly related to resilience, likely due to cognitive characteristics associated with obsessive-compulsive spectrum disorders. Individuals within this spectrum frequently demonstrate altered executive functions, including difficulties in set shifting (the ability to switch between different mental rules or tasks) and attention switching (the ability to change the focus of attention). This probably leads to a rigid cognitive style that complicates problem-solving and exacerbates anxiety in response to obsessive thoughts [26]. Lower resilience would imply a diminished capacity to adapt to stress from these obsessive thoughts, potentially leading to more severe symptoms. Importantly, as previously mentioned, one study reported an increase in resilience and cognitive flexibility three months after OCD patients began treatment with SSRIs [11]. This may indicate an additional beneficial effect of pharmacological treatment on patient well-being through modulation of resilience. Consequently, the presence of pharmacological treatment, the moderate severity of the disorder, and the fact that one-third of the participants had previously undergone CBT-ERP could explain why most participants in our study were classified as having normal resilience levels (68.89%), while only a small percentage exhibited low resilience (26.67%).

Coping strategies

Additionally, we found a weak negative correlation between seeking social support as a coping strategy and the severity of the Y-BOCS compulsive factor. Research indicates that social support is beneficial for OCD patients, promoting their readiness to change, which includes both the capability and willingness to change. The experience of sharing (encompassing how the individual feels after sharing their diagnosis, including the level of support received from others) has been identified as a predictor of readiness to change [27]. For instance, a pilot study revealed that participation in a storytelling group, where individuals shared their OCD-related experiences, fostered a greater understanding of the disorder, enhanced acceptance from family and friends, and mitigated feelings of shame and guilt associated with their symptoms [28]. These findings underscore the significance of social integration in the comprehensive treatment of OCD, which can reduce dysfunction resulting from maladaptive behaviors such as compulsions.

A weak positive association was noted between avoidance as a coping strategy and the severity of the obsessive factor of the Y-BOCS. This aligns with prior research indicating that individuals with OCD are more inclined to employ avoidance strategies and are less likely to utilize adaptive coping strategies, such as problem-solving and acceptance [8]. Additionally, research indicates that avoidance is positively associated with the severity of the Dimensional Obsessive-Compulsive Scale, particularly concerning the subscale related to unacceptable thoughts [29]. In clinical practice, these findings may support the need for supplementary interventions, in conjunction with pharmacological treatment and CBT-ERP, to promote the development of more effective coping strategies. For example, research indicates that the implementation of Acceptance and Commitment Therapy (ACT) can facilitate the use of effective coping strategies [30]. Evidence suggests a reduction in symptom severity among patients diagnosed with OCD who engage in ACT, likely due to enhanced cognitive flexibility and the adoption of positive behaviors instead of avoidant ones [31].

PTSD symptoms

Concerning trauma, six (13.33%) patients were classified as having probable PTSD. This finding is consistent with a study that estimated the comorbid presence of PTSD in 19.1% of patients diagnosed with OCD [14]. When considering the gender of the participants, no differences were identified regarding the presence and severity of PTSD. This finding stands in contrast to another study that reported a stronger association between trauma and the severity of obsessive-compulsive spectrum symptoms in women [9]. This discrepancy may be attributed to the observation that women in this study were more likely to employ problem-solving as a coping strategy. Furthermore, the severity of PTSD symptoms exhibited a positive correlation with the obsessive factor of the Y-BOCS. This correlation aligns with findings from another investigation, which noted that individuals diagnosed with both OCD and PTSD displayed greater symptom severity and poorer response to treatment compared to those with OCD alone, particularly concerning unacceptable thoughts and symmetry-related symptoms [32]. The inadequate response to unacceptable thoughts may be linked to the influence of traumatic event memories on the severity of the disorder, indicating that improvement may require the implementation of trauma-focused treatment. Some authors propose that OCD arises from a dysfunction of thinking, while a dysfunction of memory characterizes PTSD [33]. Nevertheless, it is evident that the symptomatology of these two disorders can significantly overlap and mutually influence the patient’s dysfunction. Additionally, resistance to symmetry-related symptoms may arise from magical thinking, where patients believe that maintaining symmetry and order can prevent negative events, including the recurrence of traumatic events [34]. These results may encourage psychiatrists to implement routine PTSD screening for patients diagnosed with OCD, perform a differential diagnosis between the two disorders, and consider the integration of trauma-focused interventions when PTSD symptoms negatively impact treatment outcomes.

Strengths and limitations

The primary strength of this study is that, to the authors’ knowledge, it represents the first research examining the relationship between resilience, coping strategies, and trauma in the Mexican population diagnosed with OCD. This is an important consideration given the limited scientific literature addressing factors that may influence the treatment of Mexican patients diagnosed with OCD. Mexico has distinct sociocultural aspects and specific healthcare system barriers, which differ from those found in countries where the majority of OCD research has been conducted. However, several limitations must be considered. First, participants were recruited from a specialized OCD clinic, which may limit the generalizability of the findings. This context frequently involves individuals with a higher number of comorbidities and those who have undergone multiple pharmacological trials. Second, although the sample size provided adequate statistical power for the correlations, it remains limited, as it did not permit comparisons between OCD subtypes and their relationship with the variables. Third, the presence of pharmacological treatment and CBT-ERP may have influenced many patients to exhibit normal levels of resilience, contradicting the proposed hypothesis. Furthermore, the analysis did not account for the duration of prescribed treatment, raising pertinent questions regarding its potential impact on the results. Fourth, other comorbidities, such as major depressive disorder and generalized anxiety disorder, were not assessed, despite their potential influence on the severity of OCD symptoms.

Future directions and implications of the study

Future research should aim to recruit participants from diverse settings, increase the sample size, and further evaluate the effects of both pharmacological and psychotherapeutic interventions, as well as the impact of comorbidities, to enhance the applicability of the findings. From a theoretical perspective, these findings contribute to the understanding of additional factors involved in the severity of OCD symptoms, highlighting the importance of implementing specific strategies that enhance resilience, facilitate access to social support, reduce avoidance behaviors, and assess trauma in all patients with this disorder.

## Conclusions

This study identified four key factors associated with the severity of OCD: resilience level, seeking social support as a coping strategy, avoidance behaviors, and trauma-related symptoms. These findings highlight additional elements that may influence patients’ treatment responses, quality of life, and overall prognosis. Future research should explore these variables in greater depth to develop and integrate additional interventions alongside pharmacological treatments and CBT-ERP, such as social integration and ACT. Such efforts could enhance resilience and promote the effective application of coping strategies. Finally, given the established relationship between PTSD and OCD, routine screening for trauma-related disorders is recommended, as well as the implementation of trauma-focused therapies when deemed necessary.
